# Peer Review of “COVID-19 National Football League (NFL) Injury Analysis: Follow-Up Study”

**DOI:** 10.2196/56169

**Published:** 2024-02-13

**Authors:** Felicianus Pereira

**Affiliations:** 1 Dow University of Health Sciences, Karachi, Pakistan

**Keywords:** COVID-19, injury, prevalence, adaptation, sports medicine, follow-up, training, football, epidemiology, sport, athlete, athletic, injuries

*This is the peer-review report for “COVID-19 National Football League (NFL) Injury Analysis: Follow-Up Study.”*

## Round 1 Review

### General Comments

I would like to commend the authors on performing a follow-up study [1]. This well-executed study provides a good comparison with how COVID-19 disrupted global schedules and the impact it had on the sports sector. The previous version of this study identified how a lack of training greatly increased chances of injuries in National Football League (NFL) athletes. In the current study, the authors have identified how a timely training program can reduce the injury time of athletes. A few points require clarification, such as:

1. What is the starting month of the NFL season? The COVID-19 lockdown was implemented from late March 2020 to late May 2020 (mentioned in the current study). Did this fall at the start, at the middle, or toward the end of the training phase of the athletes?

2. Were athletes provided any equipment at home, or were they recommended any training protocol by their team’s coaches (the way some clubs in the English Premier League provided gym equipment to their players at home to maintain fitness or had online practice sessions with their players)?

3. When lockdown restrictions were lifted, how much time were the athletes provided to restore their match fitness?

4. How long does it take for detraining to set in, and how quickly can athletes regain their lost fitness?

### Minor Comments

5. Change the tense of some sentences. In the introduction, use “We hypothesized” instead of “we hypothesize” and change the next line to “injury prevalence for the 2021 and 2022 seasons WOULD be lower than the 2020 NFL season...”

## Abbreviations

NFL: National Football League
